# Relative Weights of Temporal Envelope Cues in Different Frequency Regions for Mandarin Vowel, Consonant, and Lexical Tone Recognition

**DOI:** 10.3389/fnins.2021.744959

**Published:** 2021-12-02

**Authors:** Zhong Zheng, Keyi Li, Gang Feng, Yang Guo, Yinan Li, Lili Xiao, Chengqi Liu, Shouhuan He, Zhen Zhang, Di Qian, Yanmei Feng

**Affiliations:** ^1^Department of Otolaryngology-Head and Neck Surgery, Shanghai Jiao Tong University Affiliated Sixth People’s Hospital, Shanghai, China; ^2^Shanghai Key Laboratory of Sleep Disordered Breathing, Shanghai, China; ^3^Sydney Institute of Language and Commerce, Shanghai University, Shanghai, China; ^4^Department of Graduate, The First Affiliated Hospital of Jinzhou Medical University, Jinzhou, China; ^5^Ear, Nose, and Throat Institute and Otorhinolaryngology Department, Eye and ENT Hospital of Fudan University, Shanghai, China; ^6^Department of Otolaryngology, Qingpu Branch of Zhongshan Hospital Affiliated to Fudan University, Shanghai, China; ^7^Department of Otolaryngology, Shenzhen Longhua District People’s Hospital, Shenzhen, China

**Keywords:** temporal envelope cues, frequency region, Mandarin, vowel, consonant, tone

## Abstract

**Objectives:** Mandarin-speaking users of cochlear implants (CI) perform poorer than their English counterpart. This may be because present CI speech coding schemes are largely based on English. This study aims to evaluate the relative contributions of temporal envelope (E) cues to Mandarin phoneme (including vowel, and consonant) and lexical tone recognition to provide information for speech coding schemes specific to Mandarin.

**Design:** Eleven normal hearing subjects were studied using acoustic temporal E cues that were extracted from 30 continuous frequency bands between 80 and 7,562 Hz using the Hilbert transform and divided into five frequency regions. Percent-correct recognition scores were obtained with acoustic E cues presented in three, four, and five frequency regions and their relative weights calculated using the least-square approach.

**Results:** For stimuli with three, four, and five frequency regions, percent-correct scores for vowel recognition using E cues were 50.43–84.82%, 76.27–95.24%, and 96.58%, respectively; for consonant recognition 35.49–63.77%, 67.75–78.87%, and 87.87%; for lexical tone recognition 60.80–97.15%, 73.16–96.87%, and 96.73%. For frequency region 1 to frequency region 5, the mean weights in vowel recognition were 0.17, 0.31, 0.22, 0.18, and 0.12, respectively; in consonant recognition 0.10, 0.16, 0.18, 0.23, and 0.33; in lexical tone recognition 0.38, 0.18, 0.14, 0.16, and 0.14.

**Conclusion:** Regions that contributed most for vowel recognition was Region 2 (502–1,022 Hz) that contains first formant (*F*1) information; Region 5 (3,856–7,562 Hz) contributed most to consonant recognition; Region 1 (80–502 Hz) that contains fundamental frequency (F0) information contributed most to lexical tone recognition.

## Introduction

Hearing loss is a common sensory disorder and has become an important global health problem due to the increasing prevalence and its negative impact on quality of life. World Health Organization [WHO] (2020) estimates that 466 million people suffer from hearing loss, with sensorineural hearing loss (SNHL) being the most common. Cochlear implant (CI) is currently the only effective method for patients with severe-to-profound SNHL (Zeng, 2004). Plenty of past research has been conducted to figure out the best strategies for encoding speech. Roy et al. (2015) programmed with either fine structure processing or high-definition continuous interleaved sampling strategy for CI users, and found that fine structure processing strategy offers better musical sound quality discrimination for CI users with respect to fundamental frequency perception. Tabibi et al. (2020) implemented a bio-inspired coding strategy for better representation of spectral and temporal information with 11 CI users, and significantly better performance was observed for bio-inspired coding strategy compared to the advanced combination encoder strategy. Recently, there are many studies for tonal language pitch encoding. Temporal limits encoder, optimized pitch, and language strategy has recently been proposed that can provide a significant benefit to perception of speech intonation (Meng et al., 2016; Vandali et al., 2019). The mainstream CI speech processing strategies, such as advanced combination encoder (Psarros et al., 2002), SPEAK (Skinner et al., 2002), and n-of-m (Ziese et al., 2000; Buechner et al., 2009) are based on the continuous interleaved sampling strategy (Wilson et al., 1991; Boëx et al., 1996). For the continuous interleaved sampling speech processing strategy, the electrode array is successively spaced with a single stimulus, that is, only one electrode is emitting the stimulus current at a time, and the interference and diffusion of the stimulus current between two electrodes are prevented by alternating stimulation (Zeng et al., 2008). Although contemporary CI has up to 22 intracochlear electrodes, the capacity of patients to use multiple channels typically asymptotes at around 8 channels or less (Pfingst et al., 2011; Macherey and Carlyon, 2014). Therefore, even as the most successful neural implants in the world, there is still much to be studied and improved in signal processing strategies.

Speech acoustic signals can be regarded as the temporal envelope (E) cues with slow change and the temporal fine structure (TFS) information with fast change based on the Hilbert transform (Kong and Zeng, 2006). The TFS information is the pre-dominant cues for lexical tone perception in NH listeners (Xu and Pfingst, 2003) but in hearing-impaired listeners and in noise environment, envelope cue plays an increasingly important role for lexical tone perception (Wang S. et al., 2011; Qi et al., 2017). The temporal E cues represents the amplitude of the waveform changing with time phase, which usually includes the duration information and amplitude E cues of the speech signal (Kong and Zeng, 2006). Perceptual research has shown that E cues are important for speech perception in quiet conditions (Smith et al., 2002; Xu and Pfingst, 2003). Different frequency regions of speech signals contain different information with varying functions, making it necessary to evaluate the relative importance of temporal information with different frequency regions in speech recognition. Past research methods on the role of temporal information in different frequency regions for speech recognition include removing a specific spectral information (Shannon et al., 2002), correlation analysis (Apoux and Bacon, 2004), lowpass- and highpass- filtration (Ardoint and Lorenzi, 2010), and band-pass filtration (Ardoint et al., 2011).

Previous research was mostly based on English, a non-tonal language. Mandarin, a tonal and most common spoken language in the world is significantly different from English. Mandarin includes 24 finals, 23 consonants, and 4 lexical tones. The 24 finals include 6 monophthongs, 9 diphthongs, and triphthongs, and 9 nasal finals. The 23 consonants always occur as “initials.” Phonemes that include vowels and consonants are important signals for the auditory system because they have great contribution to speech intelligibility across languages (Kewley-Port et al., 2007). The four lexical tones include Lexical tone 1- (high-level), Lexical tone 2/(rising), Lexical tone 3 v (falling-rising), and Lexical tone 4 \(falling). In Mandarin, the same words with different lexical tones can represent many different meanings (Nissen et al., 2005). Previous studies have shown that compared with normal-hearing listeners, Mandarin-speaking CI users have shown poor performance in lexical tone recognition (Wei et al., 2004; Wang W. et al., 2011). One of the most important reasons is that most Chinese CI wearers have imported devices where the language processing strategy is calibrated toward non-tonal languages. Our previous studies have shown that the acoustic temporal E cues in frequency regions 1 (80–502 Hz) and 3 (1,022–1,913 Hz) significantly contributed to Mandarin sentence recognition in quiet (Guo et al., 2017). Given that speech perception involves both bottom-up and top-down processes, sentence recognition are heavily influenced by phonemic, lexical tone, and context in top-down condition (Hickok and Poeppel, 2007). This was the basis for our investigation into the different contributions of frequency regions in Mandarin phonemic and lexical tone recognition.

## Materials and Methods

### Subjects

A group of 11 listeners (5 males, 6 females) from graduates of Shanghai Jiao Tong University were recruited in this study. Their ages ranged from 22 to 27 (average = 24.6) years with no reported history of ear disease or hearing difficulty. They were all native Mandarin Chinese speakers with normal audiometric thresholds (<20 dB HL), bilaterally, at frequencies between 0.25 and 8 kHz. Pure-tone audiometric thresholds were recorded using a GSI-61 audiometer (Grason-Stadler, Madison, WI, United States) with standard audiometric procedures. All subjects had no preceding exposure to the speech materials. Before the experiment, all subjects had signed a consent form and were compensated hourly. All procedures performed in studies involving human participants were approved and in accordance with the Ethics Committee of the Sixth People’s Hospital affiliated to Shanghai Jiao Tong University (ChiCTR-ROC-17013460) and with the 1964 Declaration of Helsinki and its later amendments.

### Signal Processing

The speech test program named Angel Sound^1^ developed by Qian-Jie Fu at the House Ear Institute (Los Angeles, CA, United States) was used for Mandarin phoneme and lexical tone tests (Wu et al., 2007). All speech materials were sourced from the language database of University of Science and Technology of China, which includes the phonemes and lexical tones most frequently used in Mandarin. All the materials were recorded by one male and one female native Mandarin speaker. All speech stimuli were sampled at a 22-kHz sampling rate, without high-frequency pre-emphasis. The test ensures that only one phoneme is different. For example, to test for vowel, combinations of the same consonant and lexical tone that carry the most vowel options were selected. For lexical tone, given the maximum possibility is four, phoneme combinations fewer than four lexical tones were excluded (Wu et al., 2007). Lexical tone duration was normalized for lexical tone tokens to minimize bias on tonal perception (Jing et al., 2017). The speech materials were filtered into 30 contiguous frequency bands using zero-phase, third-order Butterworth filters (18 dB/oct slopes), ranging from 80 to 7,562 Hz (Li et al., 2016; Guo et al., 2017; Zheng et al., 2021). Each frequency band was an equivalent rectangular bandwidth for normal people, which simulates the frequency selection of normal auditory system (Glasberg and Moore, 1990). E information was extracted from each band using the Hilbert decomposition and low-pass filter at 64 Hz using third-order Butterworth filters. Then E was used to modulate the amplitude of a white noise. The envelope-modulated noise was bandpass-filtered using the same filter parameters as before. This study focuses on the parameters used in the present CI strategy in low frequency (<500 Hz), medium low frequency (500–1,000 Hz), medium frequency (1,000–2,000 Hz), medium high frequency (2,000–4,000 Hz), and high frequency (4,000–8,000 Hz) bands. Given the cut-off frequency of each frequency band is close to 500, 1,000, 2,000, 4,000, and 8,000 Hz, the modulated noise bands were summed across frequency bands to produce the frequency regions of acoustic E cues as follows: Bands 1–8, 9–13, 14–18, 19–24, and 25–30 were summed to form Frequency Regions 1–5, respectively (Table 1). To prevent subjects from using the E cues of the adjacent boundary band (Warren et al., 2004; Li et al., 2015), the frequency region containing the E cues was combined with complementary frequency regions containing noise masker that was presented at a speech-to-noise ratio of +16 dB. The speech-to-noise ratio was determined prior to signal processing using a full range of speech and noise stimuli. Masking noise was low-pass and high-pass filtered so that the final long-term power spectrum did not overlap the processed speech signals as the previous study (Ardoint et al., 2011). To investigate the role of different frequency regions for Mandarin phoneme and lexical tone recognition, the E cues from three frequency regions (10 conditions including “Region 123,” “Region 124,” “Region 125,” “Region 134,” “Region 135,” “Region 145,” “Region 234,” “Region 235,” “Region 245,” and “Region 345”), four frequency regions (five conditions including “Region 1234,” “Region 1345,” “Region 1245,” “Region 1235,” and “Region 2345”) and five frequency regions (one condition, “Region 12345”) were presented to subjects. For example, the condition of “Region 123” meant the stimulus presented to the subject contained the E cues of frequency regions 1, 2, and 3 with noise of the remaining frequency regions 4 and 5. Similarly, in the test condition of “Region 124,” the stimulus sound contains the E cues of frequency regions 1, 2, and 4 while other frequency bands (band 3 and 5) are white noise. In the test of full band region “Region 12345,” the stimulus sound contains the E cues information of all frequency bands, and there is no other noise.

**TABLE 1:** Cut-off frequency for extracting temporal envelope information in different frequency regions.

Frequency regions	Bands	Lower frequency (Hz)	Upper frequency (Hz)
1	1	80	115
	2	115	154
	3	154	198
	4	198	246
	5	246	300
	6	300	360
	7	360	427
	8	427	502
2	9	502	585
	10	585	677
	11	677	780
	12	780	894
	13	894	1,022
3	14	1,022	1,164
	15	1,164	1,322
	16	1,322	1,499
	17	1,499	1,695
	18	1,695	1,913
4	19	1,913	2,157
	20	2,157	2,428
	21	2,428	2,729
	22	2,729	3,066
	23	3,066	3,440
	24	3,440	3,856
5	25	3,856	4,321
	26	4,321	4,837
	27	4,837	5,413
	28	5,413	6,054
	29	6,054	6,767
	30	6,767	7,562

### Test Procedure

None of the subjects had participated in the perception experiments testing acoustic temporal E cues before. The experiments were conducted in a double-walled, soundproof room. All test stimuli were delivered through Sennheiser HD205 II circumaural headphones. The stimuli were determined according to the most comfortable level of the subjects, generally around 65 dB SPL.

Before the formal test, ∼30 min of practice were provided. The speech material was presented under “full Region” conditions initially, and then presented in the same way as the test condition stimulus. Feedback was given during the practice. To familiarize the subjects with the test material, they can repeatedly listen to a word indefinitely and move on after they feel they have reached a stable state.

In the formal test, we randomly selected test sounds from different conditions and allowed subjects to hear the same test sound multiple times. Subjects were required to focus on repeating the keywords as accurately as possible, and we encouraged them to guess the uncertain words. Our observation was that most participants listened to each word two or three times before moving on. Vowel and consonant recognition were measured using a 16-alternative identification paradigm. The response buttons were labeled using vowel syllables for the vowel recognition task, consonant context with common finals for the consonant recognition task. Lexical tone recognition was measured using a four-alternative identification paradigm, and “Lexical tone 1,” “Lexical tone 2,” “Lexical tone 3,” and “Lexical tone 4” for the Mandarin lexical tone recognition task. No feedback was given during the formal test. Each word was rated as correct or incorrect, and then the percentage of correct words was recorded under different conditions. Subjects can take a rest at any time to minimize fatigue during testing. The complete test time for each participant is ∼1.5–2 h.

### Least-Squares Approach

To evaluate the weight of the five frequency regions in Mandarin phoneme and lexical tone recognition using acoustic temporal E cues, we calculated the weight of each frequency region using the least-squares approach previously used in other research (Kasturi et al., 2002). The strength of each frequency region was defined as a binary value of 0 or 1, depending on whether the frequency region was presented or not. Then, the weight of each frequency region was calculated by predicting the subject’s response as a linear combination of the contribution of each frequency region. The initial weights of each subject’s five frequency regions were converted to relative weights by summing them up, and the weights of each frequency region were expressed as the initial weight divided by the sum of all five frequency regions weights. Therefore, the weights of the five frequency regions add up to 1.0 (For more details, please see Supplementary Material).

### Statistical Analysis

The Statistical Package for Social Sciences (SPSS) version 24.0 (IBM Corp., Armonk, NY, United States) was used for statistical analysis. The one-way analysis of variance (ANOVA) with repeated measures was used for the results from different test conditions for phoneme and lexical tone recognition. The *post hoc* analysis (Tukey’s test) was used for pairwise comparison. The least-squares approach was used to calculate the relative weights of the five frequency regions. The independent samples *t*-test was used to compare the relative weights of five frequency regions in Mandarin phoneme and lexical tone recognition. The figures were generated by GraphPad Prism 8.0 (GraphPad Software, San Diego, CA, United States). Statistical significance was set at *p* < 0.05.

## Results

### Scores for Mandarin Phoneme and Lexical Tone Recognition Across Conditions Using Temporal E Cues

**FIGURE 1:**
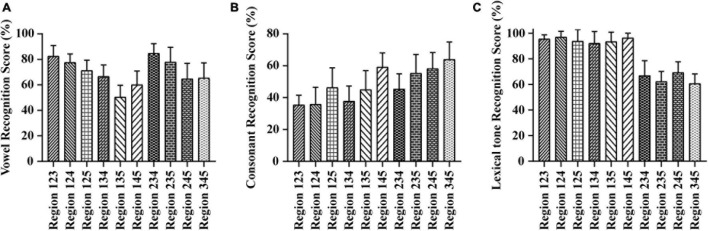
Averaged percent-correct scores for Mandarin phoneme and lexical tone recognition using acoustic temporal envelope with three frequency regions conditions. The error bars represent standard errors. **(A)** Averaged scores for Mandarin vowel recognition with envelope cues in three frequency regions conditions. **(B)** Averaged scores for Mandarin consonant recognition with envelope cues in three frequency regions conditions. **(C)** Averaged scores for Mandarin lexical tone recognition with envelope cues in three frequency regions conditions.

As shown in Figure 1A, the vowel recognition scores ranged from 50.43 to 84.82% when the E cues were presented in three frequency regions. The Region 234 condition score was the highest, ∼84.82%, while Region 135 was lowest, ∼50.43%. A one-way repeated-measures ANOVA of different test conditions with three frequency regions showed significant differences in vowel recognition scores among different frequency regions combinations [*F*_(9,100)_ = 13.559, *p* < 0.05]. The Tukey’s test revealed that the score under the Region 135 and Region 145 conditions was significantly lower than the scores under all other conditions with three frequency regions (*p* < 0.05). The consonant recognition scores ranged from 35.49 to 63.77% when the E cues were presented in three frequency regions (see in Figure 1B). The Region 345 condition score was the highest, ∼63.77%, while Region 123 was lowest, ∼35.49%. A one-way repeated-measures ANOVA of different test conditions with three frequency regions showed significant differences in consonant recognition scores among different frequency region combinations [*F*_(9,100)_ = 11.622, *p* < 0.05]. The Tukey’s test revealed that the scores obtained from conditions combined with Frequency Region 5 would be higher than those obtained from conditions combined without Region 5 (Region 123, Region 124, Region 134, and Region 234) (*p* < 0.05). The lexical tone recognition scores ranged from 60.80 to 97.15% when the E cues were presented in three frequency regions (see in Figure 1C). The Region 124 condition score was the highest, ∼97.15%, while Region 345 was lowest, ∼60.80%. A one-way repeated-measures ANOVA of different test conditions with three frequency regions showed significant differences in lexical tone recognition scores among different frequency region combinations [*F*_(9,100)_ = 46.910, *p* < 0.05]. The Tukey’s test revealed that the scores obtained from conditions combined with Frequency Region 1 would be higher than those obtained from conditions combined without Region 1 (Region 234, Region 235, Region 245, and Region 345) (*p* < 0.05).

**FIGURE 2:**
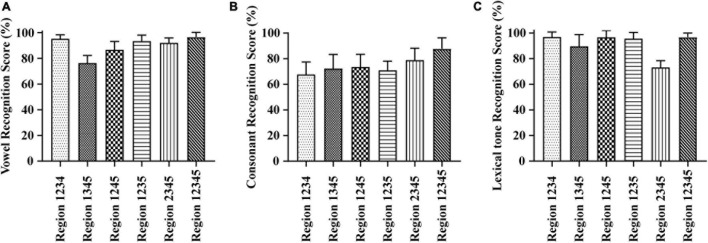
Averaged percent-correct scores for Mandarin phoneme and lexical tone recognition using acoustic temporal envelope with four and five frequency regions conditions. The error bars represent standard errors. **(A)** Averaged scores for Mandarin vowel recognition with envelope cues in four frequency regions conditions. **(B)** Averaged scores for Mandarin consonant recognition with envelope cues in four frequency regions conditions. **(C)** Averaged scores for Mandarin lexical tone recognition with envelope cues in four frequency regions conditions.

As shown in Figure 2A, the vowel recognition scores ranged from 76.27 to 96.58% when the E cues were presented in four frequency regions. The Region 1234 condition score was the highest, ∼95.24%, while Region 1345 was the lowest, ∼76.27%. When stimulus presented in full frequency regions, the score raised to 96.58%. A one-way repeated-measures ANOVA of different test conditions with four and five frequency regions showed significant differences in vowel recognition scores among different frequency region combinations [*F*_(5_,_60)_ = 27.674, *p* < 0.05]. The Tukey’s test revealed that the score under the Region 1345 condition was significantly lower than the score under all other conditions (*p* < 0.05). The consonant recognition scores ranged from 67.75 to 78.87% when the E cues were presented in four frequency regions (see in Figure 2B). The Region 2345 condition score was the highest, ∼78.87%, while Region 1234 was the lowest, ∼67.75%. When stimulus presented in full frequency regions, the score raised to 87.87%. A one-way repeated-measures ANOVA of different test conditions with four and five frequency regions showed significant differences in consonant recognition scores among different frequency region combinations [*F*_(5,60)_ = 6.462, *p* < 0.05]. The Tukey’s test revealed that the difference in the five conditions with four frequency regions was not significant (*p* = 0.063). However, the consonant recognition scores with full frequency regions were significantly higher than that in four frequency regions combinations (*p* < 0.05). The lexical tone recognition scores ranged from 73.16 to 96.87% when the E cues were presented in four frequency regions (see in Figure 2C). The Region 1234 condition score was the highest, ∼96.87%, while Region 2345 was lowest, ∼73.16%. When stimulus presented in full frequency regions, the score raised to 96.73%. A one-way repeated-measures ANOVA of different test conditions with four and five frequency regions showed significant differences in consonant recognition scores among different frequency region combinations [*F*_(5,60)_ = 30.802, *p* < 0.05]. The Tukey’s test revealed that the score under the Region 2345 condition was significantly lower than the score under all other conditions with four frequency regions (*p* < 0.05).

### Relative Weights of the Five Frequency Regions in Mandarin Phoneme and Lexical Tone Recognition

**FIGURE 3:**
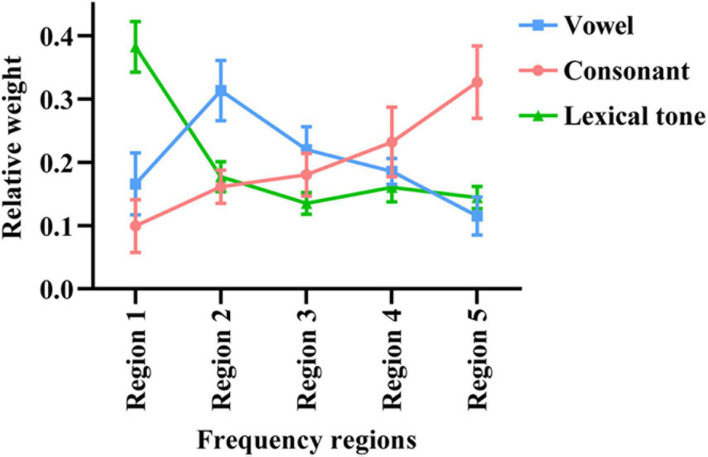
The relative weights of different frequency regions for Mandarin phoneme and lexical tone recognition using acoustic temporal envelope. The error bars represent standard errors.

As shown in Figure 3, the mean weights of frequency region 1–5 for vowel recognition were 0.17, 0.31, 0.22, 0.18, and 0.12, respectively. The one-way ANOVA showed a significant main effect of region on weight for vowel recognition [*F*_(4,50)_ = 41.117, *p* < 0.05]. The Tukey’s test revealed that the relative weight of Region 2 was highest than all other regions while the relative weight of Region 5 was lowest than all other regions (*p* < 0.05). The mean weights of frequency region 1–5 for consonant recognition were 0.10, 0.16, 0.18, 0.23, and 0.33, respectively. The one-way ANOVA showed a significant main effect of region on weight for consonant recognition [*F*_(4,50)_ = 40.459, *p* < 0.05]. The Tukey’s test revealed that the relative weight of Region 5 was highest than all other regions while the relative weight of Region 1 was lowest than all other regions (*p* < 0.05). The mean weights of frequency region 1–5 for lexical tone recognition were 0.38, 0.18, 0.14, 0.16, and 0.14, respectively. The one-way ANOVA showed a significant main effect of region on weight for lexical tone recognition [*F*_(4,50)_ = 176.725, *p* < 0.05]. The Tukey’s test revealed that the relative weight of Region 1 was highest than all other regions (*p* < 0.05).

## Discussion

This study was designed to explore the relative importance of acoustic E cues across different frequency regions for Mandarin phoneme and lexical tone recognition. Then we calculated the weight of each frequency region in Mandarin phoneme and lexical tone recognition by the least-squares approach as shown in Figure 3. Region 2 (502–1,022 Hz), Region 5 (3,856–7,562 Hz), and Region 1 (80–502 Hz) significantly contributed to Mandarin vowel, consonant, and lexical tone recognition, respectively.

Previous reports suggested that Mandarin phonemes and sentence recognition improved dramatically when the number of frequency regions increased from one to four (Fu et al., 1998), which was in line with results found in English speech recognition (Shannon et al., 1995). Our results affirm these findings, and that when presented in full region, the temporal E cues are sufficient to code for the recognition of Mandarin phoneme and lexical tone.

Vowels are important to the power of speech that is characterized by open vocal tract with sustained vocalization, low-frequency energy, and long duration (Chen et al., 2013; Chen and Chan, 2016). Sounds typically have four or five formants when it passes through the vocal tract. The first two formants determine the quality of vowels, while the last three formants determine the individual’s unique timbre and influence the individual’s vocal characteristics. When vowels are pronounced, the height of the tongue position corresponds to the first formant (F1), and the front and back of the tongue position correspond to the second formant (F2) (Lopes et al., 2018). Formant frequencies F1 and F2 have long been known to be crucial for encoding the phonetic identity of vowels (Carney et al., 2015; Fogerty, 2015). Hillenbrand et al. (1995) analyzed the data obtained from 45 men, 48 women, and 46 children and revealed that F1 (342–1,022 Hz) and F2 (910–3,081 Hz) were sufficient for vowel classification. It seems that the locations of the formants are dispersed optimally in the F1–F2 space, as described in dispersion theory (Schwartz et al., 1997). With the increase of the size of vowel systems, this dispersion leads to the consistencies among linguistic vowel systems in the appearance of vowel contrasts. While a study (Parikh and Loizou, 2005) reported that vowel recognition in noise is supported mainly by information about F1 along with some information about F2, another study (Xu et al., 2005) analyzed the information transmitted for acoustic features of vowels and found that the duration and F1 cues rather than F2 cues contributed substantially to vowel recognition. This is closer to a report (Traunmüller, 1981) that suggested the simultaneous distance between F1 and the fundamental frequency (F0) is the primary determinant of perceived vowel height. In our research, we found that the scores under the conditions without Region 2 (Region 135, Region 145, and Region 1345) were significantly lower than the score in other conditions (seen in Figures 1A, 2A). The mean weights of frequency region 1–5 for vowel recognition were 0.17, 0.31, 0.22, 0.18, and 0.12, respectively. The relative weight of Region 2 (502–1,022 Hz) was highest across all other regions (seen in Figure 3). This is consistent with previously reported study (Kasturi et al., 2002) that channels 1, 3, and 4, centered at 393, 1,037, and 1,685 Hz, respectively, received the largest weight for vowels recognition and will lead to decrease in listener’s performance if removed.

Different from vowels, consonants are characterized by complete or partial vocal tract constriction with high-frequency energy and short duration that are important to speech intelligibility (Chen et al., 2013; Chen and Chan, 2016). For consonants, many of these phonemes are characterized by rapid, instantaneous changes in amplitude, for instance, those caused by burst noise (Stevens, 2002). Therefore, high frequencies phoneme level modulation may be particularly important for conveying the consonant cues necessary for intelligibility. These high-frequency bands are characterized by having fast rate E modulations. A previous research (Fogerty, 2014) replaced consonant and vowel segments with noise matched to speech spectrum and found that consonants contain higher frequency components compared to vowels. We found that high-frequency region (3,856–7,562 Hz) of E cues plays a crucial role in consonant recognition (seen in Figure 3), and the scores obtained from conditions combined with Region 5 would be higher than those obtained from conditions combined without Region 5 (seen in Figure 1B). However, our result of relative weight for consonant recognition is different from previous findings (Kasturi et al., 2002). Here, the relative weight for the consonants was quite flat and all channels (the frequencies ranged from 300 to 4,444 Hz) are equally important for consonant recognition. In contrast, a study (Apoux and Bacon, 2008) reported that consonant recognition was not affected by removing E cues above 4 Hz in the low- and high-frequency bands, while the consonant recognition decreased as the cutoff frequency was decreased in the mid-frequency region from 16 to 4 Hz. Possible reasons for the different weight for consonant recognition include: (1) differences in speech materials, for the type of speech material may have a strong impact on the value of acoustic information (Lunner et al., 2012); (2) the different methods of processing stimuli and different cut-off frequencies used in different experiments; (3) finally and most importantly the difference may come from differences in languages. The plosive bursts in Mandarin consonants produce a strongly synchronized burst of energy across the frequency spectrum related to high frequency regions (Drullman et al., 1994).

Lexical tone is also called pitch or the height of the sound. Most ordinary sounds can be analyzed as a sum of sinusoidal components with harmonic frequencies and evoke a pitch corresponding to their F0 (Santurette and Dau, 2011). As previous studies reported, F0 cues are important for Chinese lexical tone recognition (Luo and Fu, 2004; Chen et al., 2014; Vandali et al., 2015). There are four lexical tones in Mandarin including Lexical tone 1- (high-level), Lexical tone 2/(rising), Lexical tone 3 v (falling-rising), and Lexical tone 4 \(falling). As a tonal language, the same phonetic segment carries a different meaning when produced with different lexical tones (Chen et al., 2013). Lexical tones of Mandarin have been studied by many researchers. As previous research reported, Lexical tone 1 is associated with a flat F0 contour and Lexical tone 2 with a rising F0 contour (Whalen and Xu, 1992), Lexical tone 3 has the lowest intensity and longest duration while Lexical tone 4 is usually the strongest and shortest lasting pitch (Kuo et al., 2008). Although these four lexical tones are mainly distinguished by the F0 cues, other characteristics including overall intensity and duration vary systematically with lexical tone (Kuo et al., 2008). A study (Chen et al., 2014) examined the effects of lexical tone on the intelligibility of Mandarin revealed that the F0 contour is particularly important in tonal recognition in noisy environments. Another study (Vandali et al., 2015) reported that training with a single cue (F0 and center frequency) can improve the recognition ability of pitch and timbre without other cues variations. This is similar to another finding (Luo and Fu, 2004) that found modifying the amplitude E to make it closer to the F0 contour may be an effective method to improve the lexical tone recognition of Chinese CI wearers. Our findings are consistent with these previous studies, where Region 1 (80–502 Hz) significantly contributes to Mandarin lexical tone recognition (seen in Figure 3).

There are some limitations in our study. First, the age of the participants ranged from 21 to 27, so the result has limited explanation for other age groups including the infant and the elderly. Secondly, the subjects in this study received higher education. Larger-scale studies are needed to verify the results, and for further research. Thirdly, the preliminary results our study currently obtained will be used to guide more in-depth research, however, how signal processing or stimulation strategies for future CI systems will be influenced by current results is unclear. Perhaps CI users, in addition to normal hearing listeners, could further be recruited to increase the impact of this research.

## Conclusion

(1) For Mandarin vowel recognition, Region 2 (502–1,022 Hz) which contained the first formant (*F*1) information contributed more than other regions.
(2) For Mandarin consonant recognition, Region 5 (3,856–7,562 Hz) contributed more than other regions.
(3) For Mandarin lexical tone recognition, Region 1 (80–502 Hz) which contained fundamental frequency (F0) information contributed more than other regions.

## Data Availability Statement

The original contributions presented in the study are included in the article/Supplementary Material, further inquiries can be directed to the corresponding authors.

## Ethics Statement

The studies involving human participants were reviewed and approved by the Shanghai Jiaotong University Affiliated Sixth People’s Hospital Ethics Committee. The patients/participants provided their written informed consent to participate in this study.

## Author Contributions

ZZhe, KL, ZZha, DQ, and YF: conceptualization. KL, YG, and SH: methodology. KL, GF, and YG: data curation. YL, LX, CL, and SH: investigation. ZZhe: writing—original draft preparation. ZZha, DQ, and YF: writing—review and editing. YF: funding acquisition. ZZhe and YF: resources. ZZha: supervision. All authors contributed to the article and approved the submitted version.

## Conflict of Interest

The authors declare that the research was conducted in the absence of any commercial or financial relationships that could be construed as a potential conflict of interest.

## Publisher’s Note

All claims expressed in this article are solely those of the authors and do not necessarily represent those of their affiliated organizations, or those of the publisher, the editors and the reviewers. Any product that may be evaluated in this article, or claim that may be made by its manufacturer, is not guaranteed or endorsed by the publisher.

## Footnotes

1
http://angelsound.tigerspeech.com/
