# Supplementary material for: Relative Weights of Temporal Envelope Cues in Different Frequency Regions for Mandarin Vowel, Consonant, and Lexical Tone Recognition
Source: Front Neurosci. 2021 Dec 2;15:744959. doi: 10.3389/fnins.2021.744959 (PMC8678109; doi:10.3389/fnins.2021.744959)
Supplement: Supplementary file 2 [file Data_Sheet_2.docx]

# Least-squares approach

Our approach to obtain the importance of each frequency band follows the method proposed by Kasturi et al. [1]. Results from the recognition scores for Mandarin phoneme and pitch contour were used to predict the importance or perceptual ‘‘weight’’ of each frequency region.

Weight $w_{i}$ of each region was calculated by predicting the responses of the subject as a linear combination of strength in each frequency region, i.e.,

$R_{k}=\sum_{i=1}^{5} w_{i}E_{ik}$ , (1)

where $R_{k}$ is the mean percent-correct score for condition *k,* and $E_{ik}$ is the strength of the *i*th region corresponding to condition k. The strength of each region is a binary value that can be either 0 or 1 depending on whether the frequency region was presented or not, respectively. The value of k ranges from 1 to 16 spanning all region combinations (Supplementary Table 1). Forming the prediction error $e_{k}$

$e_{k}=R_{k}-\sum_{i=1}^{5} w_{i}E_{ik}$ , (2)

Region weights can then be estimated by minimizing the sum of all the squared errors with respect to $w_{i}$ . Alternatively, Eq. (1) can be written in matrix form as

$R=EW$, (3)

where R is a 16-dimensional vector containing the mean percent-correct scores for conditions 1 to 16, E is the data matrix (16*5) consisting of the strengths of each region (Supplementary Table 1), and $W=\left[ w_{1},w_{2},w_{3},w_{4},w_{5} \right]$ is a 5-dimensional vector consisting of the desired region weights.

The above set of equations represents an over deterministic system of equations since we have 5 unknowns (the region weights) and 16 equations (one for each condition). We calculated the weights $W$ by solving the matrix equation given by (3) using a least-squares approach

$W={(E^{T}E)}^{-1}E^{T}R$ . (4)

After obtaining the solution from Eq. (4), we normalized the weights so that the sum of all the weights was equal to 1.

Supplementary Table 1. The 16 test conditions considered in this study. The strength of each frequency region was defined as a binary value of 0 or 1, depending on whether the frequency region was presented or not.

| No. | Conditions | Region 1 | Region 2 | Region 3 | Region 4 | Region 5 |
| --- | --- | --- | --- | --- | --- | --- |
| 1 | Region 123 | 1 | 1 | 1 | 0 | 0 |
| 2 | Region 124 | 1 | 1 | 0 | 1 | 0 |
| 3 | Region 125 | 1 | 1 | 0 | 0 | 1 |
| 4 | Region 134 | 1 | 0 | 1 | 1 | 0 |
| 5 | Region 135 | 1 | 0 | 1 | 0 | 1 |
| 6 | Region 145 | 1 | 0 | 0 | 1 | 1 |
| 7 | Region 234 | 0 | 1 | 1 | 1 | 0 |
| 8 | Region 235 | 0 | 1 | 1 | 0 | 1 |
| 9 | Region 245 | 0 | 1 | 0 | 1 | 1 |
| 10 | Region 345 | 0 | 0 | 1 | 1 | 1 |
| 11 | Region 1234 | 1 | 1 | 1 | 1 | 0 |
| 12 | Region 1345 | 1 | 0 | 1 | 1 | 1 |
| 13 | Region 1245 | 1 | 1 | 0 | 1 | 1 |
| 14 | Region 1235 | 1 | 1 | 1 | 0 | 1 |
| 15 | Region 2345 | 1 | 1 | 1 | 1 | 0 |
| 16 | Region 12345 | 1 | 1 | 1 | 1 | 1 |

**Reference**

1. Kasturi K, Loizou PC, Dorman M, Spahr T (2002) The intelligibility of speech with "holes" in the spectrum. The Journal of the Acoustical Society of America 112 (3 Pt 1):1102-1111. doi:10.1121/1.1498855
